# Meeting Milestones: Results of a Quality-Improvement Curriculum to Achieve Cost-Conscious Care

**DOI:** 10.1080/21614083.2018.1517572

**Published:** 2018-09-25

**Authors:** Olivia Chang, JoAnn Jordan, Neel Shah, Monica Mendiola, Anna Merport Modest, Toni Golen

**Affiliations:** a Department of Obstetrics and Gynecology, Beth Israel Deaconess Medical Center, Boston, MA, USA; b Department of Obstetrics, Gynecology and Reproductive Biology, Harvard Medical School, Boston, MA, USA

**Keywords:** Medical education, quality-improvement, residency curriculum

## Abstract

There is a lack of residency education in cost-conscious care. We implemented a costing and quality improvement (QI) curriculum to Obstetrics and Gynaecology trainees using “Time-Driven Activity-Based Costing (TDABC),” and assessed its educational impact.

The curriculum included didactic and practical portions. Pre-and post-knowledge surveys were obtained from 24 residents on self-perceived knowledge of key QI principles. Self-perceived knowledge, before and after the curriculum, was scored on a Likert scale from 0 to 5 points (0 is the least knowledge and 5 is the most knowledge). The mean scores reported an increase in knowledge of clinical guideline development (pre = 1.19 vs. post = 3.07, *p* = 0.0052); confidence in participating in QI work (pre = 1.75 vs. post = 3.42 points, *p* < 0.0001); and knowledge in communicating QI principles (pre = 1.89, post = 3.17, *p* < 0.0003). Our educational programme uses the TDABC method and the residents’ clinical experience effectively to teach residents cost-conscious care.

## Background

Studies show that cost-conscious care does not result in poor patient outcomes [1]. In academic medicine, though, formulating a comprehensive differential diagnosis and work-up has historically been preferred to a cost-conscious medical plan [2]. Consequently, there is a paucity of relevant teaching resources.

The Accreditation Council for Graduate Medical Education (ACGME) and American Board of Medical Specialties (ABMS) identified lack of training in the domain of cost-conscious care. In 2015, ACGME moved to the Next Accreditation System (NAS) and “Milestones” framework. Milestones describe the learning trajectory throughout training with the goal of demonstrating a high level of competency [3], within milestones such as “Cost-Effective Care and Patient Advocacy,” “Patient Safety and Systems Approach to Medical Errors,” and “Quality Improvement Process.”

There have been attempts to create curricula on cost-conscious care [4,5]. Some argue that these attempts may be significantly limited [6], as the results showed only a temporary effect [7]. This may be because traditional didactic-based curricula are often decoupled from the realities of clinical workflow. Other data show practice patterns acquired in residency will persist for the rest of professional life [8]. New methods of teaching adult learners that integrate an understanding of clinical workflow may be more successful in teaching cost-conscious care.

We introduced the Time-Driven Activity-Based Costing (TDABC) concept as a vehicle for learning value, cost-conscious care, and quality improvement. TDABC was developed at Harvard Business School [9]; it breaks up clinical care into individual steps using process mapping, and assigns cost to each step on a per-minute basis. Process maps provide a visual depiction of clinical workflow: specific symbols are used to represent when a decision is required, when an activity occurs, and when an outcome results. All steps and costs are visible, making it easier to identify areas that do not add value. Case studies using this approach have been linked to improved systems performance [10,11]. We hypothesised that TDABC, as part of a QI curriculum, would provide a way to teach quality improvement and cost-conscious care.

## Methods

Twenty-four female obstetrics-gynaecology residents, or physicians-in-training, participated. The residents’ post-graduate year ranged from 1 to 4. The first portion of the curriculum was the educational component led by four OB/GYN generalists, including two with particular expertise in the field of quality improvement. There were three didactic sessions that focused on the principles of process mapping, the basic skills of cost analysis, and approaches to designing a systematic process improvement intervention.

**Figure 1.**
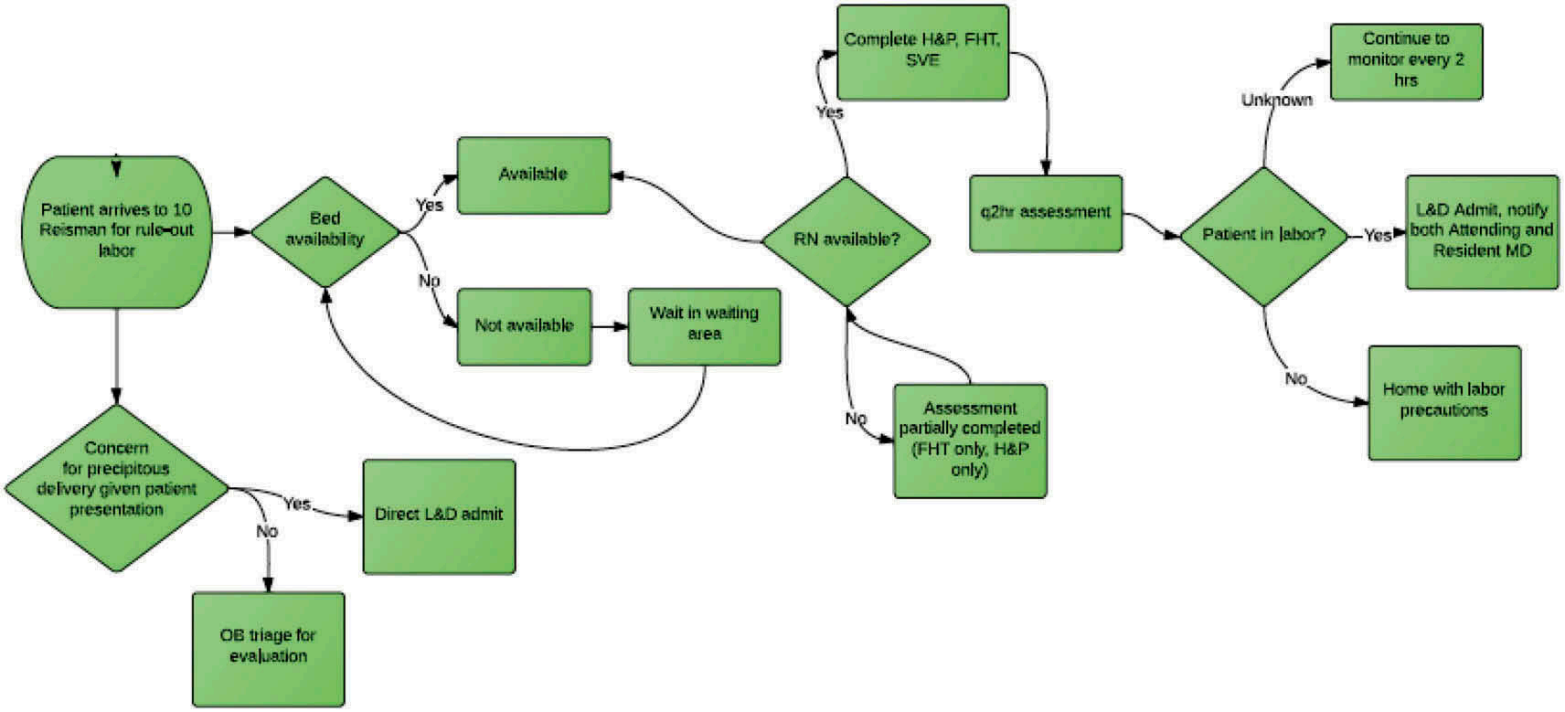
Process map for women presenting for rule-out labour.

The second portion focused on process mapping and cost accounting. The residents were separated into five teams and assigned to create process maps for vaginal birth after caesarean, spontaneous vaginal delivery and caesarean delivery. Residents created two process maps for each of these scenarios (Figure 1). The first was created from memory with the goal of learning how to create process maps. A second was made from direct observation of the process in order to improve process maps in an iterative fashion through direct observation and feedback. Maps were then scored by blinded reviewers.

In addition, trainees played a cost accounting game we created called, “The Process Is Right”. This game presented the learner with a childbirth scenario in steps and forced decisions at key points. The cost of each decision was totalled. Twenty-one residents played the game.

Residents completed pre- and post-curriculum surveys to assess understanding of value-based care (Appendix). They were asked about their knowledge on the following QI principles: root cause analysis, process mapping, adverse event reporting at BIDMC, Plan-Do-Study-Act (PDSA) cycle, run charts, clinical guideline development, confidence in participating in QI project, ability to communicate to colleague about QI principles, role of QI as part of the physician’s professional activities, difference between pricing and cost, difference between quality and value, and just culture. The knowledge scale was scored on a Likert scale from 0 to 5, with 0 as least knowledge and 5 as most knowledge.

All statistical analyses were performed using SAS 9.4 (SAS Institute Inc., Cary, NC, USA). All tests were two-sided, with *P* < 0.05 considered to be statistically significant. Data were presented as median and interquartile range (IQR). Wilcoxon signed-rank test were used to compare paired medians.

## Results

For “The Process is Right” game, the 21 residents spent median amount of $617.14, with range of $474.24–$1,426.04. The resident who won the game was able to obtain a $474.24 cost for a safe vaginal delivery.

A cross-sectional survey was completed before and after the QI curriculum. Twenty-one residents completed the pre-surveys and 21 residents completed the post-survey. Eighteen residents filled out both the pre- and post-survey (85.7%). When asked about knowledge of process maps, the average score was 1.12. After the curriculum and having had actual experience creating process maps as a team, the post-curriculum average score was 3.49, a significant improvement (< 0.0001) (Table 1). Residents reported a positive increase in knowledge on clinical guideline development, with a pre-curriculum average score of 1.19, with the post-curriculum average score of 3.07 points (*p* = 0.0052).

**Table 1.** Changes in self-perceived knowledge scales on core quality improvement principles, before and after the QI curriculum.

Factor	Pre (*n* = 18)	Post (*n* = 18)	Change (*n* = 18)	*p*-Value
Process mapping	1.12 (0.74, 2.12)*	3.49 (2.97, 4.09)	1.99 (0.90, 2.89)*	< .0001
Clinical guideline development	1.19 (0.27, 2.00)***	3.07 (2.50, 3.70)	1.63 (0.41, 3.27) ***	0.0052
Ability to identify QI opportunities in case presentations and day-to-day patient care	2.01 (1.23, 2.48) **	3.72 (3.37, 4.20)	1.38 (0.73, 2.37) **	< .0001
Confidence in participating in a QI project	1.75 (0.96, 3.05)	3.42 (3.06, 4.24)	1.44 (0.58, 3.07)	< .0001
Ability to communicate with colleagues about QI principles	1.89 (1.27, 2.30) *	3.17 (2.93, 3.48)	1.09 (0.70, 2.23) *	0.0003
Role of QI as part of a physician’s professional activities	2.00 (1.59, 2.72) **	3.42 (3.06, 4.03)	1.11 (0.79, 1.64) **	0.0002
The difference between quality and value	2.06 (1.00, 3.03) ***	3.27 (2.63, 4.04)	1.05 (0.17, 2.10) ***	0.0017

Data are presented as median and interquartile range. *Missing two responses. **Missing one response. ***Missing three responses.

When asked about their ability to identify QI opportunities in case presentations and day-to-day patient care, there was a significant improvement in knowledge with the average pre-curriculum score of 2.01 to a post-curriculum average score of 3.72 (*p* < 0.0001). The residents’ confidence in participating in a QI project increased from 1.75 to 3.42 (*p* < 0.0001). Residents also felt more knowledgeable in communicating with colleagues about QI principles (pre-curriculum average score = 1.89, post-curriculum average score = 3.17, *p* < 0.0003).

## Discussion

Residents’ extensive exposure to the clinical environment confers an understanding of hospital workflow. A resident can appreciate the minutia of the admission, inpatient and discharge process, describe impediments to care, and frequently have ideas for improvement. However, the ideas for improvement frequently arise without consideration to cost or value.

After exposure to this curriculum, residents stated that they became more aware of how they can change the distribution of resources in order to spend their time valuably. This curriculum also provides a forum for residents to devise ideas for quality improvement, again relying on their unique position and ample time on the clinical units.

Residents were able to apply their knowledge and personal experience with the cost-accounting game of “The Process is Right”. While the objective of the game was to achieve safe vaginal delivery at the lowest cost, the underlying goal is for residents to appreciate how every step of clinical decision-making does alter the total cost, which may or may not affect the outcome. The wide range of spending for the game perhaps reflects the clinical practice variation that a QI curriculum like ours can aim to resolve.

Our curriculum integrates QI into training programmes. With knowledge of quality improvement, residents will graduate with better understanding of the value of healthcare. This is relevant to residents’ careers after residency, as with new healthcare legislation in the news [12], patient satisfaction and outcomes will likely be more scrutinised.

The strength of our curriculum is that it allows residents to achieve their ACGME milestones. By including residents of all levels, our comprehensive curriculum allowed residents to achieve their milestones earlier in their training. This method of teaching cost-conscious care is novel as it combines process mapping and the cost of the individual steps of care into the science of improvement and does so in a way that trainees can readily understand it. This curriculum also gave trainees the opportunity to increase value and improve care processes.

The limitation of this project is our small sample size, and the cost-accounting portion is difficult to replicate unless the game is available. The costs are unique to our institution. However, the curriculum is adaptable for other institutions. Another limitation was that we did not have a control group for comparison as all of our residents participated in this curriculum. By introducing stewardship of cost-conscious care, we wanted all of our residents to carry this skill set into their professional careers and make an impact with their clinical decision-making.

## Conclusion

Our curriculum is an effective educational programme to increase residents’ self-perceived knowledge on core quality improvement principles. The curriculum relies on residents’ unique perspective and familiarity with workflow, integrated with the TDABC method, to design interventions to decrease cost.

## Appendix

1. Despite delivering the most expensive care in the world, adverse medical events are common in the USA. To what extent do you think each factor below contributes to adverse medical events in the USA?

______ Lack of sufficient clinical guidelines

______ Lack of compliance with existing guidelines

______ Apathetic clinicians

______ Complexity of the clinical environment

______ Medically unnecessary treatments

______ Poorly coordinated care

______ Human error

2. You are the Director of Quality Improvement. Recently, there has been a surge of infections after caesarean delivery in your department. Your hospital would like to reduce the incidence of infections. We already know that these infections were caused by common bacteria and involve patients of nearly all physicians in the department. Which of the following interventions would you do first, second and third? Please rank 1–3 in order of priority, with “1” being the action you would take first.

______ Introduce a form to document infection prevention steps for each patient

______ Meet with physicians about their infection rates

______ Observe the care steps for caesarean delivery patients

______ Review the literature to find guidelines

______ Remind physicians to pay close attention to post-op fever

______ Design an evidence-based intervention

______ Perform cultures of physicians’ hands

3. Six months have passed since the post-caesarean infections were on the rise. You have determined that the principal cause of the infections is inconsistent practice regarding choice of antibiotics before the skin incision, and you would like to target your intervention to correct this. Which approach do you think would reduce the greatest number of infections (choose one)?

- Development of an evidence-based guideline
- Mandatory pause before surgery to confirm correct choice of antibiotic
- Patient education about the importance of prophylactic antibiotics
- An electronic barrier in the pharmacy system to prevent ordering the wrong antibiotic
- Review charts and send messages to physicians when they choose the wrong antibiotic
- Post signs to remind physicians about correct antibiotic choice

4. You are quite successful as the Director of Quality Improvement, and you are handed your next project. The hospital is now concerned about patient satisfaction survey results. Specifically, your department is performing very poorly in response to the question, “Did you know the names of your doctors?” Among the following, which title best describes the project that you think would be the most effective value-added strategy for improving this score?

- Patient Satisfaction: Challenges Due to Physician Workload
- Hello My Name Is: Teaching Basic Manners to Trainees (2)
- The Art and Science of How to Spend More Time at the Bedside (3)
- Budget Request for Electronic Whiteboards to Improve Name Recognition (4)
- Nice to Meet You: Introducing a Standardised Physician Greeting Process (5)

5. You have achieved success as the Director of Quality Improvement, and news of your good work travels to Washington. You are appointed as President Obama’s Special Advisor for Health Care Reform and must now send teams of advisors to hospitals to teach doctors how to improve the quality of care. Which of the following statements is/are consistent with the goal of improving the quality of care? You may choose more than one answer.

- One of the central goals of Quality Improvement is the elimination of individual physician and nurse error.
- All improvement interventions should focus on the caregiver as the centre of the process, and benefits to the patients will follow.
- Rewards should be given to staff that participate in Quality Improvement projects, and even very small improvements should be celebrated.
- Excellent care is created by the consistent and reliable delivery of established best practices.
- When delivering complex care, leaders need to resist deviation from established guidelines, even when encountering varying caregiver skill or the evolving nature of disease.
- Command and control of care teams leads to a reduction in adverse outcomes.
- Continuous improvement depends on continuous measurement, transparency and feedback.

6. You are now in the twilight of your career and have moved on to teaching Quality Improvement to the first-year medical students at Dartmouth. It is a three-lecture series and it is the only didactic they will receive on the subject. In compiling your syllabus, which three topics would you talk to them about? Choose 3.

- Physician accountability for medical error
- The impaired physician
- Diagramming multi-step care processes
- The epidemic of obesity
- Defining harm
- How to design a Quality Improvement project
- The response to epidemics of disease
- The Plan-Do-Study-Act cycle
- The malpractice crisis

7. Please indicate how knowledgeable you feel about the following principles (slide the slider to reflect your level of knowledge on the scale).

______ Root cause analysis

______ Process mapping

______ Adverse event reporting at the hospitals

______ Plan-Do-Study-Act cycle (PDSA)

______ Run charts

______ Clinical guideline development

______ Ability to identify QI opportunities in case presentations and day-to-day patient care

______ Confidence in participating in a QI project

______ Ability to communicate with colleagues about QI principles

______ Role of QI as part of a physician’s professional activities

______ Failure mode event analysis (FMEA)

______ The difference between pricing and cost

______ The difference between quality and value

______ Human factors engineering

______ Just culture

## References

[1] Fisher ES, Bynum JP, Skinner JS. Slowing the growth of health care costs–lessons from regional variation. N Engl J Med. 2009;360(9):849–5. 19246356 10.1056/NEJMp0809794 PMC2722744

[2] Rosenbaum L, Lamas D Cents and sensitivity–teaching physicians to think about costs. N Engl J Med. 2012;367(2):99–101. 22784112 10.1056/NEJMp1205634

[3] Holmboe E, Edgar L, Hamstra S The milestones guidebook. [Cited 2016 1127] Available from: http://www.acgme.org/Portals/0/MilestonesGuidebook.pdf

[4] Lyle CB Jr, Bianchi RF, Harris JH, et al Teaching cost containment to house officers at Charlotte Memorial Hospital. J Med Educ. 1979;54(11):856–862. 501716 10.1097/00001888-197911000-00005

[5] Manheim LM, Feinglass J, Hughes R, et al Training house officers to be cost conscious. Effects of an educational intervention on charges and length of stay. Med Care. 1990;28(1):29–42. 2296215 10.1097/00005650-199001000-00005

[6] Cooke M Cost consciousness in patient care — what is medical education’s responsibility? N Engl J Med. 2010;362(14):1253–1255. 20357275 10.1056/NEJMp0911502

[7] Eisenberg JM, Williams SV Cost containment and changing physicians’ practice behavior: can the fox learn to guard the chicken coop? JAMA. 1981;246(19):2195–2201. 7197307

[8] Asch DA, Nicholson S, Srinivas S, et al Evaluating obstetrical residency programs using patient outcomes. JAMA. 2009;302(12):1277–1283. 19773562 10.1001/jama.2009.1356

[9] Kaplan RS, Porter ME How to solve the cost crisis in health care. Harv Bus Rev. 2011;89(9): 46–52, 54, 56–61 passim. 21939127

[10] Schon Klinik: measuring cost and value. Harvard business review. [cited 2014 1230]. Available from: https://hbr.org/product/schon-klinik-measuring-cost-and-value/112085-PDF-ENG

[11] Boston Children’s Hospital: measuring patient costs. Harvard business review. [cited 2014 1230]. Available from: https://hbr.org/product/boston-children-s-hospital-measuring-patient-costs/112086-PDF-ENG

[12] Office of the Legislative Counsel Compliation of patient information and affordable care act. 2010 [cited 2016 1127]. Available from: http://www.hhs.gov/sites/default/files/ppacacon.pdf

